# Serotonin: a novel bone mass controller may have implications for alveolar bone

**DOI:** 10.1186/1477-5751-12-12

**Published:** 2013-08-21

**Authors:** Carlo Galli, Guido Macaluso, Giovanni Passeri

**Affiliations:** 1 Dep. Biomedicine, Biotechnology and Translational Sciences, University of Parma, Via Gramsci 14, Parma 43126, Italy; 2 Dep. Clinical and Experimental Medicine, University of Parma, Parma, Italy

**Keywords:** Serotonin, Alveolar bone loss, Serotonin uptake inhibitors, Periodontitis

## Abstract

As recent studies highlight the importance of alternative mechanisms in the control of bone turnover, new therapeutic approaches can be envisaged for bone diseases and periodontitis-induced bone loss. Recently, it has been shown that Fluoxetine and Venlafaxine, serotonin re-uptake inhibitors commonly used as antidepressants, can positively or negatively affect bone loss in rat models of induced periodontitis. Serotonin is a neurotransmitter that can be found within specific nuclei of the central nervous system, but can also be produced in the gut and be sequestered inside platelet granules. Although it is known to be mainly involved in the control of mood, sleep, and intestinal physiology, recent evidence has pointed at far reaching effects on bone metabolism, as a mediator of the effects of Lrp5, a membrane receptor commonly associated with Wnt canonical signaling and osteoblast differentiation. Deletion of Lrp5 in mice lead to increased expression of Tryptophan Hydroxylase 1, the gut isoform of the enzyme required for serotonin synthesis, thus increasing serum levels of serotonin. Serotonin, in turn, could bind to HTR1B receptors on osteoblasts and stop their proliferation by activating PKA and CREB.

Although different groups have reported controversial results on the existence of an Lrp5-serotonin axis and the action of serotonin in bone remodeling, there is convincing evidence that serotonin modulators such as SSRIs can affect bone turnover. Consequently, the effects of this drug family on periodontal physiology should be thoroughly explored.

## Commentary

A recent study by Branco-de-Almeida [1] showed that ligature-induced periodontitis in rats could be ameliorated by Fluoxetine, a selective serotonin re-uptake inhibitor (SSRI), a class of molecules that can increase serotonin levels by inhibiting its clearance inside synapses and are commonly used as antidepressant and as an effective treatment for mood disorders [2]. Carvalho et al., however, showed in this Journal that Venlafaxine, a member of the same drug class, increased bone loss in a rat model of induced periodontitis [3]. These studies raise the question whether and how SSRIs, and therefore serotonin, may affect alveolar bone and the outcome of periodontitis. The issue is of the utmost importance because it could help elucidate poorly known aspects of periodontal pathophysiology in the context of the ongoing debate in bone metabolism, paving the way, if possible, to new therapeutic approaches. As the relation of serotonin and bone is still fiercely debated, the same controversy that has been troubling the bone field seems to be heading for periodontics.

**Figure 1:**
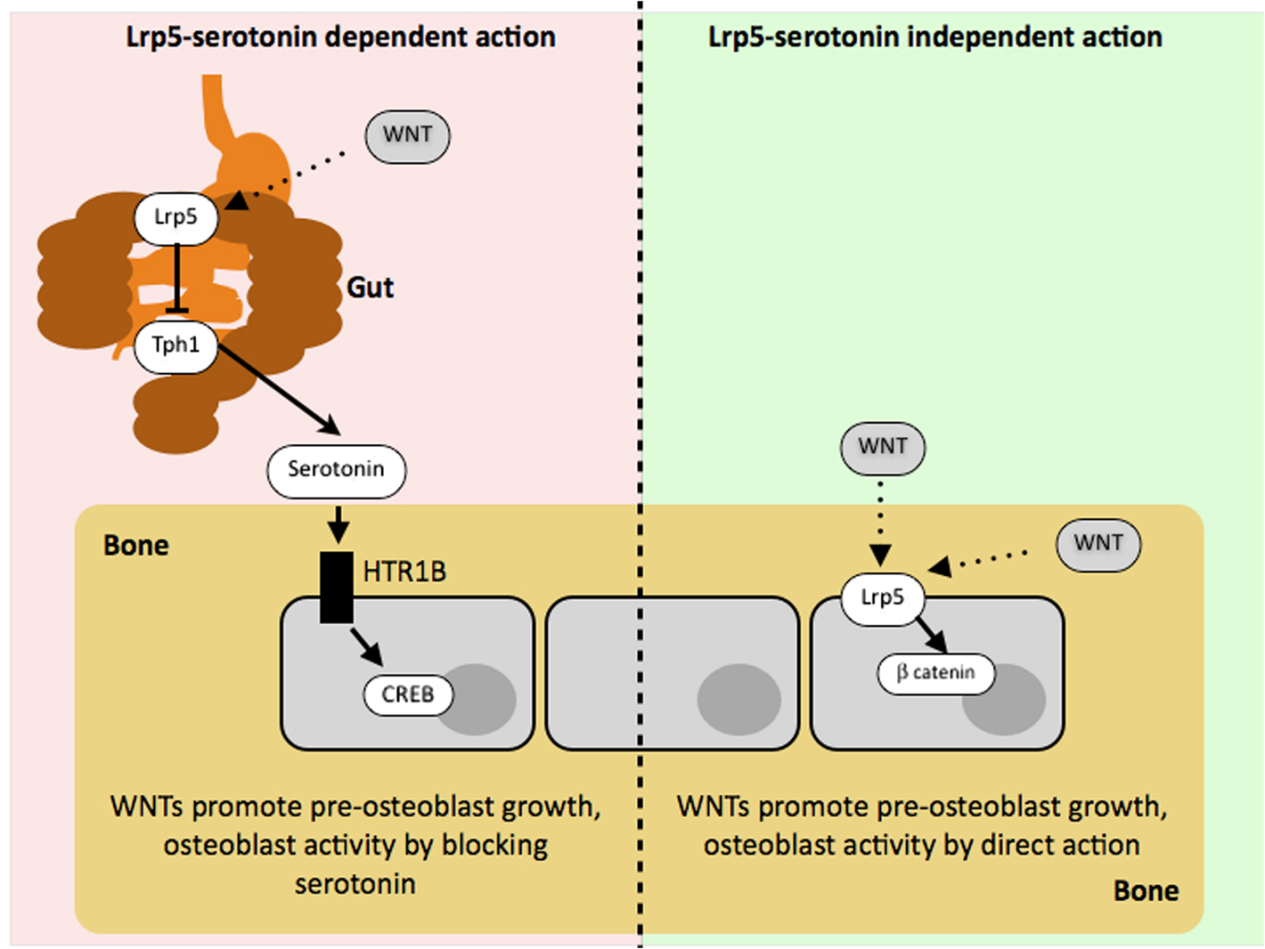
**Diagram depicting the two alternative models for Lrp5 action.** According to the former, “central”, serotonin-mediated model (left-hand side), activation of Lrp5 inhibits Tryptophan Hydroxylase 1 (Tph1) and reduces circulating levels of Serotonin, thus decreasing its inhibitory effect on osteoblasts via its HTR1B receptor. According to the “peripheral” serotonin-independent model (right-hand side), Lrp5 acts directly on osteoblasts initiating the canonical Wnt signal cascade and inducing beta catenin translocation to the nucleus and activation of its transcriptional program.

## Serotonin

Serotonin, or 5-hydroxytryptamine (5-HT), is a monoamine produced within the central nervous system, mostly in neurons located in the raphe nuclei [4], which send numerous projections to different brain regions, such as the striatum, hippocampus and frontal cortex [5]. Serotonin acts as a neurotransmitter by being released into the synaptic cleft, where it binds to post synaptic receptors. DA transporters (DAT) and 5-HT transporters (5-HTT) collect serotonin from the synaptic cleft and store it in cytoplasmic vescicles in presynaptic neurons, a process commonly referred to as re-uptake, thus regulating the duration of the stimulus. The serotoninergic transmission has a broad spectrum of effects and has been associated to neural development [6], numerous behavioral and mood disorders [7-11] and to central modulation of pain [12]. Serotonin, however, can also be synthesized by heterochromaffin cells in the gut, where it regulates gastrointestinal function [13], by endothelial cells in the lung [14] and can be found sequestered inside platelet granules [15,16]. As serotonin cannot cross the hematoencephalic barrier, it forms two physically and functionally separated pools, the former inside the central nervous system and the latter in the peripheral body. Although its best known roles are the control of mood, sleep/wake rhythm, peristalsis and mucus secretion, a big but not uncontroversial amount of evidence has been recently reported showing that serotonin may actually exert far reaching effects on bone.

A growing amount of evidence in the literature has also shown that the use of SSRIs is significantly associated to increased risk of fracture [17-23], to increased levels of bone resorption markers [24] and administrating SSRIs during pregnancy is associated to shorter length and smaller head circumference in newborns, albeit with unaffected bone quality [25]. Moreover, the frequency of serotonin transporter gene 5-HTTVNTR polymorphism was observed to be higher in osteoporotic patients [26] and 5-HTT polymorphism has been associated to BMD changes after SSRI treatment [21]. Noteworthy, a cross-sectional study by Costa et al. reported an association between aggressive periodontitis and serotonin transporter 5-HTTLPR polymorphism, which has reduced transcriptional efficiency and is associated with lower serotonin re-uptake, in the Brazilian population [27].

## The Wnt connection

Our understanding of the role of serotonin has however been considerably increased by studying genetic diseases in human and the effect of alterations in the LRP5 protein. LRP5 is a membrane protein that is commonly believed to function by dimerizing with transmembrane receptors of the Frizzled family and activating the canonical WNT signaling pathway upon binding to WNT Growth Factors [28-30]. The activation of the canonical pathway requires the recruitment of Disheveled (Dvl) [31,32], which rescues beta catenin from degradation. Beta catenin can be normally found in two pools within the cell, either bound to cadherins in cell-to-cell junctions or in the cytoplasm, where it is sequestered by a destruction complex that targets it for proteosomal degradation [33-36]. Once beta catenin is released, following activation of the receptor complex, it can translocate to the nucleus and bind to a member of the T cell factor/lymphoid enhancer factor (TCF/Lef1) transcription factor family [37,38]. The canonical WNT signaling is required for osteoblast differentiation, bone formation and even bone maintenance through osteoclast inhibition [39-41].

It is well known that loss of function or gain of function mutations in the Lrp5 gene lead respectively to a low or high bone mass phenotype, as clinically observed in Osteoporosis Pseudoglioma Syndrome (OPPG) and the High-bone-mass Syndrome (HBM) [42,43] and the majority of researchers interpret this as the result of Wnt signaling inhibition in osteoblasts. Yadav et al. however showed that deletion of Lrp5 gene in mice lead to increased expression of Tryptophan Hydroxylase 1 (Tph1), the gut isoform that is required for 5-HT synthesis [44]. As a consequence, serum levels of serotonin were increased in Lrp5 knock-out homozygous and heterozygous mice. Their findings are in agreement with numerous clinical observations in OPPG and HBM patients [45-47] and suggest that altered LRP5 functionality may be acting on bone at a distance by regulating circulating levels of serotonin, which would then bind to specific receptors on bone cells, and not directly by controlling Wnt signaling in bone cells, as commonly maintained (Figure 1). Strikingly, ex vivo experiments revealed that 50 mM 5-HT could indeed stop osteoblast proliferation by binding to the HTR1B receptor and activating PKA and CREB transcription factor [44], although previous independent results showed that lower (0.1 mM) doses of serotonin could enhance the proliferation of human primary osteoblasts in vitro [48], suggesting a possible dose-dependent effect. According to Yadav et al. tryptophan-free diet could rescue the bone phenotype in Lrp5^−/−^ mice and, importantly, conditional Lrp5 deletion in the gut increased bone mass [44]. In agreement with these observations, Laporta et al. reported that feeding 5-hydroxy-l-tryptophan to rats from day 13 of pregnancy through day 9 of lactation increased total serum and milk calcium concentrations, osteoclast numbers in bone and bone mRNA levels for resorption markers [49]. Other researchers however have gathered solid evidence supporting the idea that Lrp5 does act locally in bone and does not require serotonin. Niziolek et al. reported an Lrp5 gain-of -function mutation associated with high bone mass in mice without concomitant alterations in circulating serotonin levels [50]. Most strikingly, the same group also showed that global Tph1 deletion did not affect bone mass in their mouse model, in strong contrast to Yadav’s data [51], and Gustafsson et al. reported that administration of 5 mg/kg/day serotonin subcutaneously for 3 months increased Bone Mineral Density and cortical thickness, although reduced trabecular thickness [52].

The pre-clinical observations in rats by Carvalho et al. and Branco-de-Almeida et al. reflect this controversy and do not manage to resolve the conundrum on whether and how 5-HT acts on bone and periodontium. Unfortunately, neither Carvalho nor Branco-de-Almeida considered the role of serotonin in planning their studies and interpreting their results and as a consequence they did not measure serotonin levels in untreated and treated rats. However, measuring serum serotonin is arguably not an easy task and this could also help explain the discordant results in the literature [53]. Carvalho tested both a low (10 mg/kg/day) and a high (50 mg/kg/day) dose of Venlafaxine per os, whereas only a single but high dose of Fluoxetine (20 mg/kg/day) was used by Branco-de-Almeida, similarly orally administered, so, although it is reasonable to assume that serotonin levels were affected in both studies, it is hard to guess the real extent of it. Moreover, Fluoxetine has been shown to directly reduce osteoblast proliferation and decrease the Osteoprotegerin/Receptor Activator of Nuclear kB Factor Ligand (RANKL) ratio in vitro, thus possibly promoting osteoclastogenesis [48]. Consistently with these results, a 6 month low dose (5 mg/kg/day) treatment with Fluoxetine in rats has been shown to reduce trabecular thickness and increase endocortical bone volume [54]. The dose of Fluoxetine tested by Branco-de-Almeida et al. was on the high end of the doses commonly used to suppress serotonin production in rats [55,56], about twice as high as the maximum recommended human dose (MRHD) [54], and was based on a previous work highlighting its anti-inflammatory properties [57]. It is actually possible that this high dose of Fluoxetine may be activating alternative immunomodulatory or antiinflammatory pathways that are responsible for the authors’ observations and that are overcoming the effects on serotonin metabolism. Indeed Branco-de-Almeida et al. showed that Fluoxetine inhibited IL-1β and COX-2 mRNA and metalloprotease (MMP) 9 activity in their model, and, consistently with these findings, it has been reported that 5 mg/kg i.p Fluoxetine can reduce the expression of MMP 2 and 9 in rat hippocampus [58] and 10 mg/kg i.p. Fluoxetine inhibits the expression of MMP 2, 9 and 12 after spinal cord injury in mouse [59]. A recent study found that SSRIs can differentially control osteoclast and osteoblast viability, apoptosis and activity [60]. Fluoxetine in particular proved to affect preosteoclast viability to a greater extent than other drugs of this class, and this could help explain its effect on periodontitis. Furthermore, it cannot be ruled out that at least part of the positive effects of Fluoxetine on periodontitis could be explained through its action on the central nervous system, where it has been shown that Fluoxetine can increase Tph expression [56,61]. Strikingly, Yadav et al. showed that deletion of the brain stem specific Tryptophan Hydroxylase 2 (Tph2) isoform, lead to a reduction in bone mass in mice, apparently activating an alternative and opposite mechanism to the one mediating the effects of the gut Tph isoform [62]. Both the amount of serotonin and its localization appear therefore important for its net effect on bone. Different drugs at different doses might even prevalently act on or have higher affinity for one isoform of the enzyme and the net effect observed on bone could be the result of the systemic suppression of both enzymes.

The controversy is therefore still open and further studies that address this issue in detail are sorely needed.

## Perspectives

One of the most exciting aspects of bone mass control by serotonin is the possibility to positively affect bone formation at a distance without acting directly on bone cells, and thus the possibility of a novel therapeutic target to improve the outcome of periodontal disease and alveolar bone regenerative techniques. As of today, a novel compound, LP533401, has been generated and tested in rodents [63,64]. LP533401 can selectively inhibit Tph1 when administered per os, and significantly reduce serotonin levels in blood without passing the hematoencephalic barrier and affect brain’s serotonin concentration. It has been shown that LP533401 can increase bone mass and reduce ovariectomy-induced bone loss in rodents [63,64], even if its active enantiomer, LP923941, was independently proven to be unable to do so [50]. It has not been investigated yet whether LP533401 can affect alveolar bone.

Another equally fascinating aspect of the relation between serotonin and Lrp5 is the possibility, if confirmed, to elucidate some hitherto poorly known regulatory mechanisms in bone physiology. A central dogma of bone biology is that bone tissue is constantly remodeled by teams of dedicated cells that resorb bone, the osteoclasts, and cells that form new tissue, the osteoblasts. These cells act in a tightly coordinated fashion so that bone formation and bone resorption are coupled, because these two processes occur simultaneously and affect each other. It has been shown that osteoclasts can mobilize sequestered osteogenic factors such as TGFb1 from the mineralized matrix during resorption, stimulating osteoblasts and presumably contributing to sustain bone formation in the osteoclastic lacuna [65]. It is also known that cells of the osteoblastic lineage can produce RANKL at different stages of differentiation [66,67], thus controlling osteoclast formation and survival. This poses significant limits to anabolic therapies, which could significantly benefit from the possibility to differentially control these processes, according to a patient’s clinical needs. Noteworthy, Lrp5 deletion in mice, a genotype recapitulating the human OPPG syndrome [68], is mainly a bone formation phenotype, whereas hypomorphism of LRP6, a closely related molecule, has been shown to lead to low bone mass due to increased bone resorption [69]. A better understanding of this difference and the different signaling cascades that are located downstream, such as possibly serotonin, could indeed unravel the secret to uncoupling bone formation and resorption, with huge therapeutical benefits.

Although the available results in rodents are just preliminary, they are undoubtedly captivating and are an open invitation to further investigate the role of serotonin not only in bone physiology but also on periodontal physiology and pathology.

## Competing interests

The authors declare that they have no competing interests. Work was funded by Research Grant from ITI Foundation and Fondazione Cariparma.

## Authors’ contributions

GC conceived the manuscript and drafted it. PG helped GC drafting the manuscript and acted as consultant on bone physiology. MG acted as consultant on serotonin physiology and SSRI antidepressants. All authors read and approved the final manuscript.

## Authors’ information

GC DDS, PhD is mainly interested in alveolar bone regeneration and new approaches to promote the healing of bone defects.

PG MD, PhD is a bone specialist who focuses on bone loss and osteoporosis.

MG MD, DDS, PhD was trained as a neurologist and a periodontologist. His main research focus is oral neurophysiology, TMD and sleep disorders.

## References

[B1] Branco-de-Almeida LS Franco GC Castro ML Dos Santos JG Anbinder AL Cortelli SC Kajiya M Kawai T Rosalen PL Fluoxetine inhibits inflammatory response and bone loss in a rat model of ligature-induced periodontitis J Periodontol 2012 83 664 671 10.1902/jop.2011.110370 21966942 PMC3364595

[B2] Hoyer D Hannon JP Martin GR Molecular, pharmacological and functional diversity of 5-HT receptors Pharmacol Biochem Behav 2002 71 533 554 10.1016/S0091-3057(01)00746-8 11888546

[B3] Carvalho RS De Souza CM Neves JC Holanda-Pinto SA Pinto LM Brito GA De Andrade GM Effect of venlafaxine on bone loss associated with ligature-induced periodontitis in wistar rats J Negat Results Biomed 2010 9 3 10.1186/1477-5751-9-3 20546603 PMC2895576

[B4] Jensen P Farago AF Awatramani RB Scott MM Deneris ES Dymecki SM Redefining the serotonergic system by genetic lineage Nat Neurosci 2008 11 417 419 10.1038/nn2050 18344997 PMC2897136

[B5] Beaulieu JM A role for Akt and glycogen synthase kinase-3 as integrators of dopamine and serotonin neurotransmission in mental health J Psychiatry Neurosc 2012 37 7 16 10.1503/jpn.110011 PMC3244494 21711983

[B6] Daws LC Gould GG Ontogeny and regulation of the serotonin transporter: providing insights into human disorders Pharmacol Ther 2011 131 61 79 10.1016/j.pharmthera.2011.03.013 21447358 PMC3131109

[B7] Veenstra-VanderWeele J Anderson GM Cook EH Jr Pharmacogenetics and the serotonin system: initial studies and future directions Eur J Pharmacol 2000 410 165 181 10.1016/S0014-2999(00)00814-1 11134668

[B8] Virkkunen M Linnoila M Serotonin in early onset, male alcoholics with violent behaviour Ann Med 1990 22 327 331 10.3109/07853899009147915 2291840

[B9] Kishi T Yoshimura R Fukuo Y Okochi T Matsunaga S Umene-Nakano W Nakamura J Serretti A Correll CU Kane JM Iwata N The serotonin 1A receptor gene confer susceptibility to mood disorders: results from an extended meta-analysis of patients with major depression and bipolar disorder Eur Arch Psychiatry Clin Neurosci 2013 263 2 105 118 10.1007/s00406-012-0337-4 22752684

[B10] Oberlander TF Fetal serotonin signaling: setting pathways for early childhood development and behavior J Adolesc Health 2012 51 S9 S16 10.1016/j.jadohealth.2012.04.009 22794534

[B11] Albert PR Benkelfat C Descarries L The neurobiology of depression--revisiting the serotonin hypothesis. I. Cellular and molecular mechanisms Philos Trans R Soc Lond B Biol Sci 2011 367 2378 2381 22826338 10.1098/rstb.2012.0190 PMC3405681

[B12] Lee YC Nassikas NJ Clauw DJ The role of the central nervous system in the generation and maintenance of chronic pain in rheumatoid arthritis, osteoarthritis and fibromyalgia Arthritis Res Ther 2011 13 211 10.1186/ar3306 21542893 PMC3132050

[B13] Crowell MD Role of serotonin in the pathophysiology of the irritable bowel syndrome Br J Pharmacol 2004 141 1285 1293 10.1038/sj.bjp.0705762 15100164 PMC1574906

[B14] Abid S Houssaini A Chevarin C Marcos E Tissot CM Gary-Bobo G Wan F Mouraret N Amsellem V Dubois-Rande JL Inhibition of Gut- and lung-derived serotonin attenuates pulmonary hypertension in mice Am J Physiol Lung Cell Mol Physiol 2012 303 6 L500 L508 10.1152/ajplung.00049.2012 22797248

[B15] Jedlitschky G Greinacher A Kroemer HK Transporters in human platelets: physiologic function and impact for pharmacotherapy Blood 2012 119 3394 3402 10.1182/blood-2011-09-336933 22337717

[B16] De Abajo FJ Effects of selective serotonin reuptake inhibitors on platelet function: mechanisms, clinical outcomes and implications for use in elderly patients Drugs Aging 2011 28 345 367 10.2165/11589340-000000000-00000 21542658

[B17] Rabenda V Nicolet D Beaudart C Bruyere O Reginster JY Relationship between use of antidepressants and risk of fractures: a meta-analysis Osteoporos Int 2013 24 121 137 10.1007/s00198-012-2015-9 22638709

[B18] Zucker I Chodick G Grunhaus L Raz R Shalev V Adherence to treatment with selective serotonin reuptake inhibitors and the risk for fractures and bone loss: a population-based cohort study CNS Drugs 2012 26 537 547 10.2165/11633300-000000000-00000 22612695

[B19] Chau K Atkinson SA Taylor VH Are selective serotonin reuptake inhibitors a secondary cause of low bone density? J Osteoporos 2012 323061 10.1155/2012/323061 PMC3306899 22496984

[B20] Eom CS Lee HK Ye S Park SM Cho KH Use of selective serotonin reuptake inhibitors and risk of fracture: a systematic review and meta-analysis J Bone Miner Res 2012 27 1186 1195 10.1002/jbmr.1554 22258738

[B21] Calarge CA Ellingrod VL Zimmerman B Bliziotes MM Schlechte JA Variants of the serotonin transporter gene, selective serotonin reuptake inhibitors, and bone mineral density in risperidone-treated boys: a reanalysis of data from a cross-sectional study with emphasis on pharmacogenetics J Clin Psychiatry 2011 72 1685 1690 10.4088/JCP.10m06198 22244026 PMC3653135

[B22] Wu Q Bencaz AF Hentz JG Crowell MD Selective serotonin reuptake inhibitor treatment and risk of fractures: a meta-analysis of cohort and case–control studies Osteoporos Int 2012 23 1 365 375 10.1007/s00198-011-1778-8 21904950

[B23] Bakken MS Engeland A Engesaeter LB Ranhoff AH Hunskaar S Ruths S Increased risk of hip fracture among older people using antidepressant drugs: data from the Norwegian prescription database and the Norwegian Hip fracture registry Age Ageing 2013 Epub ahead of print 10.1093/ageing/aft009 23438446

[B24] Shea ML Garfield LD Teitelbaum S Civitelli R Mulsant BH Reynolds CF 3rd Dixon D Dore P Lenze EJ Serotonin-norepinephrine reuptake inhibitor therapy in late-life depression is associated with increased marker of bone resorption Osteoporos Int 2013 25 5 1741 1749 23358607 10.1007/s00198-012-2170-z PMC4066460

[B25] Dubnov-Raz G Hemila H Vurembrand Y Kuint J Maayan-Metzger A Maternal use of selective serotonin reuptake inhibitors during pregnancy and neonatal bone density Early Hum Dev 2012 88 3 191 194 10.1016/j.earlhumdev.2011.08.005 21890289

[B26] Ferreira JT Levy PQ Marinho CR Bicho MP Mascarenhas MR Association of serotonin transporter gene polymorphism 5HTTVNTR with osteoporosis Acta Reumatol Port 2011 36 1 14 19 21483275

[B27] Costa JE Gomes CC Cota LO Pataro AL Silva JF Gomez RS Costa FO Polymorphism in the promoter region of the gene for 5-HTT in individuals with aggressive periodontitis J Oral Sci 2008 50 193 198 10.2334/josnusd.50.193 18587210

[B28] van Amerongen R Mikels A Nusse R Alternative wnt signaling is initiated by distinct receptors Sci Signal 2008 1 re9 10.1126/scisignal.135re9 18765832

[B29] Yang-Snyder J Miller JR Brown JD Lai CJ Moon RT A frizzled homolog functions in a vertebrate Wnt signaling pathway Curr Biol 1996 6 1302 1306 10.1016/S0960-9822(02)70716-1 8939578

[B30] Moon RT Brown JD Yang-Snyder JA Miller JR Structurally related receptors and antagonists compete for secreted Wnt ligands Cell 1997 88 725 728 10.1016/S0092-8674(00)81915-7 9118212

[B31] Gordon MD Nusse R Wnt signaling: multiple pathways, multiple receptors, and multiple transcription factors J Biol Chem 2006 281 22429 22433 10.1074/jbc.R600015200 16793760

[B32] Leonard JD Ettensohn CA Analysis of dishevelled localization and function in the early sea urchin embryo Dev Biol 2007 306 50 65 10.1016/j.ydbio.2007.02.041 17433285 PMC2697034

[B33] Angers S Moon RT Proximal events in Wnt signal transduction Nat Rev Mol Cell Biol 2009 10 7 468 477 19536106 10.1038/nrm2717

[B34] Clevers H Wnt/beta-catenin signaling in development and disease Cell 2006 127 469 480 10.1016/j.cell.2006.10.018 17081971

[B35] Verheyen EM Gottardi CJ Regulation of Wnt/beta-catenin signaling by protein kinases Dev Dyn 2010 239 1 34 44 19623618 10.1002/dvdy.22019 PMC3173947

[B36] Roberts DM Pronobis MI Poulton JS Waldmann JD Stephenson EM Hanna S Peifer M Deconstructing the sscatenin destruction complex: mechanistic roles for the tumor suppressor APC in regulating Wnt signaling Mol Biol Cell 2011 22 1845 1863 10.1091/mbc.E10-11-0871 21471006 PMC3103401

[B37] Mosimann C Hausmann G Basler K Beta-catenin hits chromatin: regulation of Wnt target gene activation Nat Rev Mol Cell Biol 2009 10 276 286 19305417 10.1038/nrm2654

[B38] Mao CD Byers SW Cell-context dependent TCF/LEF expression and function: alternative tales of repression, de-repression and activation potentials Crit Rev Eukaryot Gene Expr 2011 21 207 236 10.1615/CritRevEukarGeneExpr.v21.i3.10 22111711 PMC3434703

[B39] Krishnan V Bryant HU Macdougald OA Regulation of bone mass by Wnt signaling J Clin Invest 2006 116 1202 1209 10.1172/JCI28551 16670761 PMC1451219

[B40] Rodda SJ McMahon AP Distinct roles for hedgehog and canonical Wnt signaling in specification, differentiation and maintenance of osteoblast progenitors Development 2006 133 3231 3244 10.1242/dev.02480 16854976

[B41] Glass DA 2nd Bialek P Ahn JD Starbuck M Patel MS Clevers H Taketo MM Long F McMahon AP Lang RA Karsenty G Canonical Wnt signaling in differentiated osteoblasts controls osteoclast differentiation Dev Cell 2005 8 751 764 10.1016/j.devcel.2005.02.017 15866165

[B42] Gong Y Slee RB Fukai N Rawadi G Roman-Roman S Reginato AM Wang H Cundy T Glorieux FH Lev D LDL receptor-related protein 5 (LRP5) affects bone accrual and eye development Cell 2001 107 513 523 10.1016/S0092-8674(01)00571-2 11719191

[B43] Boyden LM Mao J Belsky J Mitzner L Farhi A Mitnick MA Wu D Insogna K Lifton RP High bone density due to a mutation in LDL-receptor-related protein 5 N Engl J Med 2002 346 1513 1521 10.1056/NEJMoa013444 12015390

[B44] Yadav VK Ryu JH Suda N Tanaka KF Gingrich JA Schutz G Glorieux FH Chiang CY Zajac JD Insogna KL Lrp5 Controls bone formation by inhibiting serotonin synthesis in the duodenum Cell 2008 135 825 837 10.1016/j.cell.2008.09.059 19041748 PMC2614332

[B45] Yadav VK Arantes HP Barros ER Lazaretti-Castro M Ducy P Genetic analysis of Lrp5 function in osteoblast progenitors Calcif Tissue Int 2010 86 382 388 10.1007/s00223-010-9350-7 20333369

[B46] Saarinen A Saukkonen T Kivela T Lahtinen U Laine C Somer M Toiviainen-Salo S Cole WG Lehesjoki AE Makitie O Low density lipoprotein receptor-related protein 5 (LRP5) mutations and osteoporosis, impaired glucose metabolism and hypercholesterolaemia Clin Endocrinol (Oxf) 2010 72 481 488 10.1111/j.1365-2265.2009.03680.x 19673927

[B47] Frost M Andersen TE Yadav V Brixen K Karsenty G Kassem M Patients with high-bone-mass phenotype owing to Lrp5-T253I mutation have low plasma levels of serotonin J Bone Miner Res 2010 25 673 675 10.1002/jbmr.44 20200960

[B48] Gustafsson BI Thommesen L Stunes AK Tommeras K Westbroek I Waldum HL Slordahl K Tamburstuen MV Reseland JE Syversen U Serotonin and fluoxetine modulate bone cell function in vitro J Cell Biochem 2006 98 139 151 10.1002/jcb.20734 16408289

[B49] Laporta J Peters TL Weaver SR Merriman KE Hernandez LL Feeding 5-hydroxy-l-tryptophan during the transition from pregnancy to lactation increases calcium mobilization from bone in rats Domest Anim Endocrinol 2013 44 4 176 184 10.1016/j.domaniend.2013.01.005 23433710

[B50] Niziolek PJ Farmer TL Cui Y Turner CH Warman ML Robling AG High-bone-mass-producing mutations in the Wnt signaling pathway result in distinct skeletal phenotypes Bone 2011 49 5 1010 1019 10.1016/j.bone.2011.07.034 21855668 PMC3412139

[B51] Cui Y Niziolek PJ MacDonald BT Zylstra CR Alenina N Robinson DR Zhong Z Matthes S Jacobsen CM Conlon RA Lrp5 Functions in bone to regulate bone mass Nat Med 2011 17 684 691 10.1038/nm.2388 21602802 PMC3113461

[B52] Gustafsson BI Westbroek I Waarsing JH Waldum H Solligard E Brunsvik A Dimmen S van Leeuwen JP Weinans H Syversen U Long-term serotonin administration leads to higher bone mineral density, affects bone architecture, and leads to higher femoral bone stiffness in rats J Cell Biochem 2006 97 1283 1291 10.1002/jcb.20733 16329113

[B53] Goltzman D LRP5, Serotonin, and bone: complexity, contradictions, and conundrums J Bone Miner Res 2011 26 1997 2001 10.1002/jbmr.462 21713997

[B54] Westbroek I Waarsing JH van Leeuwen JP Waldum H Reseland JE Weinans H Syversen U Gustafsson BI Long-term fluoxetine administration does not result in major changes in bone architecture and strength in growing rats J Cell Biochem 2007 101 360 368 10.1002/jcb.21177 17163489

[B55] Abdel-Satera KA Abdel-Daiem WM Sayyed Bakheet M The gender difference of selective serotonin reuptake inhibitor, fluoxetine in adult rats with stress-induced gastric ulcer Eur J Pharmacol 2012 688 1-3 42 48 10.1016/j.ejphar.2012.04.019 22546225

[B56] Choi MR Hwang S Park GM Jung KH Kim SH Das ND Chai YG Effect of fluoxetine on the expression of tryptophan hydroxylase and 14-3-3 protein in the dorsal raphe nucleus and hippocampus of rat J Chem Neuroanat 2012 43 96 102 10.1016/j.jchemneu.2012.01.001 22285725

[B57] Roumestan C Michel A Bichon F Portet K Detoc M Henriquet C Jaffuel D Mathieu M Anti-inflammatory properties of desipramine and fluoxetine Respir Res 2007 8 35 10.1186/1465-9921-8-35 17477857 PMC1876225

[B58] Benekareddy M Mehrotra P Kulkarni VA Ramakrishnan P Dias BG Vaidya VA Antidepressant treatments regulate matrix metalloproteinases-2 and −9 (MMP-2/MMP-9) and tissue inhibitors of the metalloproteinases (TIMPS 1–4) in the adult rat hippocampus Synapse 2008 62 590 600 10.1002/syn.20529 18509851

[B59] Lee JY Kim HS Choi HY Oh TH Yune TY Fluoxetine inhibits matrix metalloprotease activation and prevents disruption of blood-spinal cord barrier after spinal cord injury Brain 2012 135 2375 2389 10.1093/brain/aws171 22798270

[B60] Hodge JM Wang Y Berk M Collier FM Fernandes TJ Constable MJ Pasco JA Dodd S Nicholson GC Kennedy RL Williams LJ Selective serotonin reuptake inhibitors inhibit human osteoclast and osteoblast formation and function Biol Psychiatry 2012 Epub ahead of print 10.1016/j.biopsych.2012.11.003 23260229

[B61] Baik SY Jung KH Choi MR Yang BH Kim SH Lee JS Oh DY Choi IG Chung H Chai YG Fluoxetine-induced up-regulation of 14-3-3zeta and tryptophan hydroxylase levels in RBL-2H3 cells Neurosci Lett 2005 374 53 57 10.1016/j.neulet.2004.10.047 15631896

[B62] Yadav VK Oury F Suda N Liu ZW Gao XB Confavreux C Klemenhagen KC Tanaka KF Gingrich JA Guo XE A serotonin-dependent mechanism explains the leptin regulation of bone mass, appetite, and energy expenditure Cell 2009 138 976 989 10.1016/j.cell.2009.06.051 19737523 PMC2768582

[B63] Yadav VK Balaji S Suresh PS Liu XS Lu X Li Z Guo XE Mann JJ Balapure AK Gershon MD Pharmacological inhibition of gut-derived serotonin synthesis is a potential bone anabolic treatment for osteoporosis Nat Med 2010 16 308 312 10.1038/nm.2098 20139991 PMC2836724

[B64] Inose H Zhou B Yadav VK Guo XE Karsenty G Ducy P Efficacy of serotonin inhibition in mouse models of bone loss J Bone Miner Res 2011 26 2002 2011 10.1002/jbmr.439 21608033

[B65] Tang Y Wu X Lei W Pang L Wan C Shi Z Zhao L Nagy TR Peng X Hu J TGF-beta1-induced migration of bone mesenchymal stem cells couples bone resorption with formation Nat Med 2009 15 757 765 10.1038/nm.1979 19584867 PMC2727637

[B66] Galli C Fu Q Wang W Olsen BR Manolagas SC Jilka RL O'Brien CA Commitment to the osteoblast lineage is not required for RANKL gene expression J Biol Chem 2009 284 12654 12662 10.1074/jbc.M806628200 19279010 PMC2675994

[B67] Xiong J Onal M Jilka RL Weinstein RS Manolagas SC O'Brien CA Matrix-embedded cells control osteoclast formation Nat Med 2012 17 1235 1241 21909103 10.1038/nm.2448 PMC3192296

[B68] Kato M Patel MS Levasseur R Lobov I Chang BH Glass DA 2nd Hartmann C Li L Hwang TH Brayton CF Cbfa1-Independent decrease in osteoblast proliferation, osteopenia, and persistent embryonic eye vascularization in mice deficient in Lrp5, a Wnt coreceptor J Cell Biol 2002 157 303 314 10.1083/jcb.200201089 11956231 PMC2199263

[B69] Kubota T Michigami T Sakaguchi N Kokubu C Suzuki A Namba N Sakai N Nakajima S Imai K Ozono K Lrp6 Hypomorphic mutation affects bone mass through bone resorption in mice and impairs interaction with mesd J Bone Miner Res 2008 23 1661 1671 10.1359/jbmr.080512 18505367

